# Treatment of anastomotic leak after esophagectomy: insights of an international case vignette survey and expert discussions

**DOI:** 10.1093/dote/doac020

**Published:** 2022-04-12

**Authors:** Sander Ubels, Merel Lubbers, Moniek H P Verstegen, Stefan A W Bouwense, Elke van Daele, Lorenzo Ferri, Suzanne S Gisbertz, Ewen A Griffiths, Peter Grimminger, George Hanna, Michal Hubka, Simon Law, Donald Low, Misha Luyer, Robert E Merritt, Christopher Morse, Carmen L Mueller, Grard A P Nieuwenhuijzen, Magnus Nilsson, John V Reynolds, Ulysses Ribeiro, Riccardo Rosati, Yaxing Shen, Bas P L Wijnhoven, Bastiaan R Klarenbeek, Frans van Workum, Camiel Rosman

**Affiliations:** Department of Surgery, Radboud Institute of Health Sciences, Radboud University Medical Center, Nijmegen, the Netherlands; Department of Surgery, ZGT Hospital Group Twente, Almelo, The Netherlands; Department of Surgery, Radboud Institute of Health Sciences, Radboud University Medical Center, Nijmegen, the Netherlands; Department of Surgery, Maastricht University Medical Center, Maastricht, The Netherlands; Department of Surgery, Ghent University Hospital, Ghent, Belgium; Department of Surgery, McGill University Health Centre, Montreal General Hospital, Montreal, Quebec, Canada; Department of Surgery, Amsterdam UMC, University of Amsterdam, Cancer Center Amsterdam, Amsterdam, The Netherlands; Department of Upper Gastrointestinal Surgery, University Hospitals Birmingham NHS Foundation Trust, Queen Elizabeth Hospital, Birmingham, UK; Department of Surgery, University Medical Center Mainz, Mainz, Germany; Department of Surgery, Imperial College, London, UK; Department of Thoracic Surgery, Virginia Mason Medical Center, Seattle, SE USA; Department of Surgery, Queen Mary Hospital, Hong Kong, China; Department of Thoracic Surgery, Virginia Mason Medical Center, Seattle, SE USA; Department of Surgery, Catharina Hospital, Eindhoven, The Netherlands; Department of Surgery, Ohio State University - Wexner Medical Center, Columbus, OH, USA; Department of Thoracic Surgery, Massachusetts General Hospital, Boston, MA, USA; Department of Surgery, McGill University Health Centre, Montreal General Hospital, Montreal, Quebec, Canada; Department of Surgery, Catharina Hospital, Eindhoven, The Netherlands; Department of Surgery, Department of Upper Abdominal Diseases, CLINTEC, Karolinska Institutet, Karolinska University Hospital, Stockholm, Sweden; Department of Surgery, Trinity St. James's Cancer Institute, Dublin, Ireland; Department of Gastroenterology, University of Sao Paulo, Sao Paulo, Brazil; Department of Gastrointestinal Surgery, San Raffaele Hospital IRCCS, Milan, Italy; Department of Thoracic Surgery, Zhongshan Hospital, Fudan University, Shanghai, China; Department of Surgery, Erasmus University Medical Centre, Rotterdam, The Netherlands; Department of Surgery, Radboud Institute of Health Sciences, Radboud University Medical Center, Nijmegen, the Netherlands; Department of Surgery, Radboud Institute of Health Sciences, Radboud University Medical Center, Nijmegen, the Netherlands; Department of Surgery, Canisius-Wilhelmina Hospital, Nijmegen, The Netherlands; Department of Surgery, Radboud Institute of Health Sciences, Radboud University Medical Center, Nijmegen, the Netherlands

**Keywords:** Esophageal Cancer, Esophagectomy, Anastomotic Leak, Survey, Focus Group

## Abstract

Anastomotic leak (AL) is a severe complication after esophagectomy. Clinical presentation of AL is diverse and there is large practice variation regarding treatment of AL. This study aimed to explore different AL treatment strategies and their underlying rationale. This mixed-methods study consisted of an international survey among upper gastro-intestinal (GI) surgeons and focus groups with expert upper GI surgeons. The survey included 10 case vignettes and data sources were integrated after separate analysis. The survey was completed by 188 respondents (completion rate 69%) and 6 focus groups were conducted with 20 international experts. Prevention of mortality was the most important goal of primary treatment. Goals of secondary treatment were to promote tissue healing, return to oral feeding and safe hospital discharge. There was substantial variation in the preferred treatment principles (e.g. drainage or defect closure) and modalities (e.g. stent or endoVAC) within different presentations of AL. Patients with local symptoms were treated by supportive means only or by non-surgical drainage and/or defect closure. Drainage was routinely performed in patients with intrathoracic collections and often combined with defect closure. Patients with conduit necrosis were predominantly treated by resection and reconstruction of the anastomosis or by esophageal diversion. This mixed-methods study shows that overall treatment strategies for AL are determined by vitality of the conduit and presence of intrathoracic collections. There is large variation in preferred treatment principles and modalities. Future research may investigate optimal treatment for specific AL presentations and aim to develop consensus-based treatment guidelines for AL after esophagectomy.

## INTRODUCTION

Anastomotic leak (AL) is a potentially life-threatening complication after esophagectomy, with an incidence of 10–20%.^1^^,^^2^ AL is associated with high mortality, post-operative morbidity, prolonged hospital admission and reduced quality of life.^1^^,^^3^ Patients with AL can present with various signs and symptoms which may also differ in severity. Hence, various treatment strategies for AL have been suggested ranging from supportive care to invasive approaches.^4–6^ Currently, there is a large variation in clinical practice,^7^ and detailed insight in the strategies and rationales currently used for treatment of AL is lacking.

Previous studies focused largely on different single treatment modalities, their indications and outcomes.^5^^,^^8–12^ For example, endoscopic stent placement has been suggested for AL with a defect smaller than 30% of the anastomotic circumference and endoscopic vacuum-assisted closure (endoVAC) has been suggested for sealed-off cavities smaller than 4 cm.^11^^,^^12^ However, AL treatment strategies are usually multimodal and management approaches are often determined largely by personal experience and availability of resources. Moreover, many studies have overlooked the diverse clinical presentation of AL, whereas treatment may need to be tailored accordingly.

A focus on general treatment strategies and principles, rather than individual modalities, may stimulate discussion amongst surgeons and give guidance to what strategies can be advised in which patient. Three treatment principles can be distinguished by their physiological mechanism: drainage of fluid collections, closure of the anastomotic defect (e.g. stent placement, surgical revision or esophageal diversion) and supportive interventions (e.g. antibiotics and/or feeding support). Treatment strategies may comprise a combination of these principles and may be achieved by using multiple modalities.

The aim of this study was to gain insight in current strategies applied in AL treatment and their rationale. This study did not seek to develop consensus, rather, this study used a mixed-methods approach to promote deeper understanding of treatment strategies used in current practice. This could provide a framework to improve future studies on treatment of AL after esophagectomy.

## METHODS

### Study design

A mixed-methods study was conducted to explore treatment strategies for AL and their rationales. Mixed-methods research purposefully combines quantitative and qualitative methods to answer questions that cannot be answered by either one alone and provides the opportunity for further exploration or deepening of understanding.^13–16^ The current study focused on surgeons performing resections for esophageal cancer, as they have a central role in management of patients with AL. An international case vignette survey was conducted to assess how surgeons treat patients with AL after esophagectomy according to their local practice. Qualitative focus groups with expert upper gastro-intestinal (GI) surgeons were conducted to further explore and interpret findings of the quantitative survey by complementarity.^17–20^ The study was exempt from ethical review by an institutional review board according to Dutch law and was conducted in accordance with the Good Reporting of A Mixed-Methods Study (GRAMMS) guidelines.^21^

### Survey

An open online survey was developed and consisted of two parts: a brief questionnaire and 10 case vignettes (i.e. clinical cases). The case vignettes were designed to cover the broad spectrum of clinical presentations of AL (Supplement 1). The vignettes differed according to location of the anastomosis, presence of fluid collections, presence of organ failure and vitality of the gastric conduit (Table 1). In each case vignette, respondents were asked to choose their preferred initial treatment strategy, consisting of one or multiple treatment principles (i.e. drainage, defect closure and/or supportive treatment), and subsequently choose their preferred modalities to effectuate the strategy. The survey was developed and tested together with esophageal surgeons from different countries to ensure relevance of the case vignettes and applicability of the survey in different practices and regions.

**Table 1:** Summary of case vignettes

**Case**	**Anastomosis**	**Fluid collections**	**Conduit condition**	**Organ failure**
**1**	Cervical	None	Viable	No
**2**	Cervical	Cervical	Viable	No
**3**	Intrathoracic	None	Viable	No
**4**	Intrathoracic	Mediastinal	Viable	No
**5**	Intrathoracic	Pleural	Viable	No
**6**	Intrathoracic	Mediastinal + Pleural	Viable	No
**7**	Intrathoracic	Mediastinal + Pleural^*^	Viable	No
**8**	Intrathoracic	Mediastinal + Pleural	Viable	Yes
**9**	Cervical	Mediastinal + Pleural	Viable	No
**10**	Cervical	Mediastinal	Necrotic	Yes

^*^Inadequate drain placement

The survey was distributed to all surgical members of the International Society for Diseases of the Esophagus (ISDE), European Society for Diseases of the Esophagus and Dutch Upper GI Cancer Group, and was distributed through the network of the Oesophago-Gastric Anastomosis Audit (OGAA) and TENTACLE—Esophagus study by email and Twitter. Only surgeons performing esophageal resections were allowed to participate. The survey was opened on 11 December 2020, one reminder was sent and then it was closed on 19 February 2021. Responses were gathered anonymously via online survey software (www.surveymonkey.com).

### Focus groups

Focus groups were conducted to provide a more detailed exploration and interpretation of the survey data by expert upper GI surgeons. The focus group outline was designed to cover the topics of the survey to promote integration during analysis (Supplement 2): goals of treatment, overall strategies, influence of intrathoracic fluid collections, vitality of the conduit, location of the anastomosis and organ failure.^15^ Expert upper GI surgeons were identified based on the following criteria: working in a high-volume center (≥ 60 resections/year), large personal experience with esophageal surgery (≥ 200 resections) and/or substantial scientific work on esophageal surgery or AL. Experts were invited by purposeful sampling and members of international societies (e.g. ISDE) and international (research) initiatives (e.g. EsoData, Minimally invasive Oesophagectomy ThinkTank, OGAA, TENTACLE—Esophagus) were selected and invited to ensure representation of different geographical regions.^14^^,^^22^

The total number of intended focus groups was six to gain a diverse understanding of AL treatment and reach data saturation.^23^ To promote discussion in an online setting, three to four expert upper GI surgeons were invited per session.^24^^,^^25^ Experts were asked to complete the survey before the focus groups. The focus groups were moderated by three esophageal surgeons (C.R., F.vW., B.K.), and the meeting was rehearsed to ensure standardization. The focus groups were organized in January 2021 using videoconferencing (https://zoom.com/), lasted up to 90 minutes and were recorded after obtaining consent of participants.

After each focus group, key findings and reflections were discussed and noted by the attending study team (C.R., B.K., F.vW., S.U.).^26^^,^^27^ An anonymized summary of each meeting was drafted after reexamining the recordings and written notes. The summary was sent to the participants to check the accuracy and validity of the content.^28^ After revising the meeting summaries, an overall summary was drafted to identify overlapping themes and areas of discordance. Literal quotes of individual experts participating in the focus groups were gathered and anonymized during analysis. Using literal quotes is a common practice in mixed-methods and qualitative research: quotes enhance the reliability of studies, provide evidence for the interpretation of the researcher and offer illustration or explanation.^29^^,^^30^

### Analysis

To integrate findings of the survey and focus groups, key findings of both data sources were discussed within the study team. Integration at the level of writing was performed using a *weaving approach*, by reporting qualitative and quantitative findings together per theme (e.g. treatment goals, intrathoracic collections, cervical versus intrathoracic leaks, organ failure).^15^ Continuous survey data were presented as median with interquartile range (IQR) and were compared using Mann–Whitney U test. Categorical variables were described as counts (percentages) and were compared using Chi-squared test. Differences regarding chosen treatment principles between case vignettes (e.g. cervical vs. intrathoracic anastomosis) were assessed using Mid-p McNemar test.^31^ The partially overlapping samples z-test was used to assess differences in the chosen modalities.^32^ A *P*-value below 0.05 was considered statistically significant. Analysis was performed in Statistical Package for the Social Sciences (SPSS) version 24 (IBM Corp. Version 24.0. Armonk, NY) and R version 3.6.3 using packages ‘exact2x2’ and ‘Partiallyoverlapping’.

## RESULTS

### Participants

**Fig. 1:**
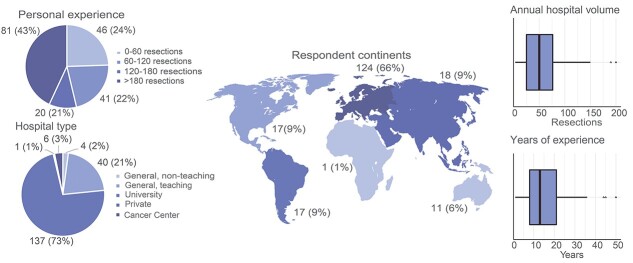
Characteristics of survey respondents.

The survey was completed by 188 out of 271 surgeons who participated (69%). Most respondents were from Europe (66%) and worked in a university hospital (73%) (Fig. 1). The median time to complete the survey was 23 minutes (IQR 16–40).

Twenty international experts from 11 countries across Europe, North America, South America and Asia participated in the focus groups, out of the 26 invited international experts (Supplementary Table S1). Six focus groups were conducted, of which one had only two participants due to an unforeseen drop-out.

### Goals of AL treatment

Prevention of mortality, reduction of morbidity and maintaining quality of life were the three most important goals of AL treatment for the majority of survey respondents (81%, 79% and 71%, respectively) (Table 2).

**Table 2:** Questionnaire responses

**Survey questions**	**Survey answers**	***n* = 188**
***Esophagectomy***		
Most commonly applied location for the anastomosis in case of a distal tumor?	Intrathoracic anastomosis Cervical anastomosis	135 (72) 53 (28)
Most commonly applied surgical approach for esophagectomy?	Transhiatal Transthoracic Unknown	19 (10) 163 (87) 6 (3)
Most commonly applied surgical technique?	Open esophagectomy hMIE, laparoscopic hMIE, thoracoscopic tMIE RAMIE	44 (23) 44 (23) 15 (8) 73 (39) 12 (7)
Most commonly performed feeding access?	Feeding jejunostomy Nasojejunal feeding tube Total parenteral nutrition IV fluids only Direct oral feeding None Other	100 (53) 28 (15) 19 (10) 7 (4) 25 (13) 3 (2) 6 (3)
***Anastomotic leak***		
Do you have a local treatment protocol for AL after esophagectomy in your hospital?	Yes No	92 (49) 96 (51)
Which treatment goals are you aiming when treating a patient with AL? Rank the treatment goals from most^1^ to least important^5^ *Median (percentage)*	Prevention of mortality Prevention or reduction of morbidity Maintaining quality of life Reducing hospital length of stay Reduction of costs	1 (81) 2 (79) 3 (71) 4 (68) 5 (81)
Please check all therapeutic modalities available in your hospital	Ultrasound-guided drainage CT-scan-guided drainage Endoscopic drainage Endoscopic stent placement endoVAC Endoscopic clipping Minimal invasive surgical treatment Open surgical treatment	173 (93) 175 (93) 163 (87) 175 (93) 120 (64) 153 (81) 168 (89) 179 (95)
What would best describe your routine treatment strategy in patients with AL?	Direct surgical intervention Conservative or minimally invasive step-up Other	6 (3) 166 (88) 16 (9)
Is antibiotic therapy indicated routinely in patients with AL?	Yes No	169 (90) 19 (10)
Is antifungal therapy indicated routinely in patients with AL?	Yes No	101 (54) 87 (46)
Do you think there is a fundamental difference in the treatment of patients with cervical AL after transthoracic (McKeown) vs transhiatal (Orringer) esophagectomy?	Yes No	53 (28) 135 (72)
Do you think there is a fundamental difference in the treatment of patients with cervical AL vs intrathoracic leak after transthoracic esophagectomy?	Yes No	145 (77) 43 (23)
How do you treat patients with AL and an ischemic gastric conduit?	Similar to patients with AL with a well-perfused conduit Similar to patients with gastric conduit necrosis Separate clinical group	45 (24) 73 (39) 70 (37)
What is your dietary prescription for patients with AL?	No restrictions (full diet) Liquids Only water Nil per mouth Dependent of the leak characteristics	2 (1) 7 (4) 27 (14) 93 (50) 59 (31)
If indicated, what is your preferred route for nutritional support for patients with AL?	Enteral, nasojejunal feeding tube Enteral, surgical jejunostomy TPN	49 (26) 123 (65) 16 (9)

Data are presented as number (percentage) or median (Interquartile Rate), unless stated otherwise.

AL, anastomotic leak; hMIE, hybrid minimally invasive esophagectomy; tMIE, totally minimally invasive esophagectomy; RAMIE, robot-assisted minimally invasive esophagectomy; IV, intravenous; TPN, total parenteral nutrition; CT, computed tomography; endoVAC, Endoscopic Vacuum-assisted Closure;

Similar goals emerged during focus group discussions, but experts distinguished between goals for primary and secondary treatment (i.e. once a patient is stabilized). The unanimous opinion of the experts was that the goal of primary treatment is to prevent mortality by management or prevention of sepsis. For secondary treatment, different goals were noted: to promote anastomotic healing, advance oral feeding, facilitate safe discharge and prevent recontamination.

### Treatment strategies

Most respondents (88%) used a minimally invasive step-up treatment strategy and approximately half of the respondents had a local treatment protocol for AL. Most treatment modalities were widely available, but availability of endoVAC was least commonly reported (64%) (Table 3).

Experts described various treatment strategies, of which many were staged strategies in line with the different goals to be achieved (Supplementary Tables S2 and S3). For primary treatment, drainage of fluid collections was thought to be the most important treatment principle in order to prevent mortality and secondary complications (e.g. airway fistula or major hemorrhage). For secondary treatment, defect closure was regarded as the main treatment principle and stent or endoVAC was performed routinely by some experts. However, the support and rationale for defect closure were much debated and not all experts rated defect closure as essential: different experts highlighted that they did not regularly used defect closure, as a leak may heal by itself.

In addition, the need for drainage during stent treatment was a controversial topic. Different experts stressed that drainage should be performed to prevent abscess formation, whilst others saw no need for drainage during stent treatment.

In general, different experts noted that, in absence of robust scientific evidence, their specific strategy was influenced by their institutional experience. Whilst the treatment goals were largely similar between experts, the principles and modalities that experts used varied substantially. However, experts highlighted that the treatment modalities were of inferior importance than the treatment strategy consisting of (multiple) treatment principles.

### Fluid collections

Sixty percent of survey respondents would perform drain placement (e.g. wound opening) in case of a cervical AL without fluid collections compared with 94% in case of a cervical fluid collection (case 1 vs. case 2, *P* < 0.001). For these confined cervical leaks, 93% of the respondents would not perform defect closure (*P* = 1.000). In patients with an intrathoracic leak without fluid collections (case 3), 32% of the surgeons would perform drain placement (e.g. at the anastomosis) compared with 81% of the surgeons in case of mediastinal collections (case 4) (*P* < 0.001) and 98% of the surgeons in case of pleural collections (case 5) (*P* < 0.001). Defect closure was performed less often in patients without fluid collections compared with patients with mediastinal or pleural fluid collections (case 1 vs. 4, 39% vs. 47%, *P* = 0.009 and case 1 vs. case 5, 39% vs. 48%, *P* = 0.006).

In patients with pleural collections, drainage was performed more often compared with mediastinal collections (case 5 vs. case 4, 99% vs. 81%, *P* < 0.001), and reoperation was the preferred modality more often (26% vs. 13%, *P* < 0.001). In these cases, no difference was found in the number of respondents that performed defect closure (case 5 vs. case 4, 48 vs. 47%, *P* = 0.71), or in the number of surgical defect closures (10% vs. 6%, *P* = 0.097).

Experts described similar strategies: patients without fluid collections were sometimes treated with supportive measures only, using antibiotics and feeding support. Nevertheless, some experts preferred defect closure or drain placement (e.g. through the anastomosis) to prevent formation of collections.

In patients with fluid collections, experts would routinely perform drainage. Nonsurgical modalities were preferred, but the modality would be chosen based on the location of the collection, degree of contamination and local expertise.

Some experts routinely used endoVAC, which was advocated to promote tissue healing and combine drainage with defect closure. Differences between mediastinal and pleural collections were noted by the experts: mediastinal cavities may sometimes drain internally spontaneously through the defect, and pleural collections were regarded as more severe if the collections were loculated (i.e. empyema).

**Table 3:** Treatment strategies for different case vignettes

**Case**	**Drainage** ***n* (%)**	**Defect closure** ***n* (%)**	**Supportive interventions** ***n* (%)**
Case 1: Cervical anastomosis, no collections	112 (60)	13 (7)	132 (70)
Case 2: Cervical anastomosis, cervical collection	177 (94)	13 (7)	114 (61)
Case 3: Intrathoracic anastomosis, no collections	60 (32)	73 (39)	147 (78)
Case 4: Intrathoracic anastomosis, mediastinal collections	152 (81)	88 (47)	125 (67)
Case 5: Intrathoracic anastomosis, pleural collections	185 (98)	90 (48)	127 (68)
Case 6: Intrathoracic anastomosis, Mediastinal and pleural collections	183 (97)	101 (54)	129 (69)
Case 7: Intrathoracic anastomosis, mediastinal and pleural collections, inadequate drainage	169 (89)	106 (56)	126 (67)
Case 8: Intrathoracic anastomosis, MOF, mediastinal and pleural collections	180 (96)	105 (56)	130 (69)
Case 9: Cervical anastomosis, mediastinal and pleural collections	185 (98)	65 (35)	125 (67)
Case 10: Cervical anastomosis, MOF, mediastinal collections, necrotic gastric conduit.	140 (75)	135 (72)	129 (69)

MOF, multiorgan failure

### Cervical versus intrathoracic leaks

The majority of respondents (77%) thought that there is a difference between treatment of AL after McKeown and Ivor Lewis esophagectomy. In absence of fluid collections, drainage was performed more often in cervical leaks (case 1 vs. case 3, 60% vs. 32%, *P* < 0.001) and defect closure was performed more often in intrathoracic leaks (case 3 vs. case 1, 39% vs. 7%, *P* < 0.001). In case of mediastinal and pleural collections, most respondents would perform drainage in both intrathoracic and cervical AL (case 6 vs. case 9, 99% vs. 98%, *P* = 0.375) but defect closure was performed less often in cervical AL (case 6 vs. case 9, 35% vs. 54%, *P* < 0.001).

Experts explained that, in absence of intrathoracic collections, cervical AL were thought to be drained more easily by opening of the cervical wound, whereas an intrathoracic leak may require endoscopy or image-guided techniques. However, the importance of drainage was similar for both leak sites. In case of intrathoracic fluid collections, treatment of cervical AL was thought to be similar to intrathoracic AL. Regarding defect closure, different experts questioned whether stent placement or endoVAC therapy was feasible and tolerated in cervical AL, and complications (e.g. migration or erosion) were thought to be more common.

### Conduit ischemia and necrosis

In case of conduit necrosis, drainage was performed less frequently compared to patients with a viable conduit (case 10 vs. case 8, 75% vs. 96%, *P* < 0.001), but defect closure was performed more often (case 10 vs. case 8, 72% vs. 56%, *P* < 0.001). In patients with necrosis, 80% of defect closures were performed by surgical diversion of the esophagus, whereas in patients with a viable conduit 75% of defect closures were performed using endoscopic techniques (*P* < 0.001).

Experts classified ischemia and necrosis as similar entities and remarked that their treatment strategy was determined by the extent of ischemia/necrosis. Small proportions of conduit ischemia/necrosis were deemed salvageable, and these patients were treated similar to patients with a viable conduit by different experts. However, patients with overall conduit ischemia/necrosis were often treated by surgical defect closure: the conduit would be resected, and reanastomosis or esophageal diversion would be performed depending on the remaining conduit length and patient condition. All experts considered diversion a last resort, given the burden for patients.

### Multiorgan failure

Drainage and defect closure were applied similarly in patients with or without multiorgan failure (case 6 vs. case 8, drainage 97 vs. 96%, *P* = 0.34, defect closure 54% vs. 56%, *P* = 0.473). However, in patients with multiorgan failure, drainage and defect closure were more often performed surgically (drainage 44% vs. 53%, *P* = 0.01, defect closure 12% vs. 25%, *P* < 0.001).

Similarly, experts of the focus groups often reserved drainage by reoperation for patients with sepsis and organ failure. Surgical drainage was thought to provide superior control of sepsis compared to non-surgical techniques in case of multiorgan failure. Moreover, some experts combined a reoperation for drainage with surgical defect closure.

## DISCUSSION

Prevention of mortality emerged as the most important goal of AL treatment, which was primarily achieved by drainage. Promoting anastomotic healing, oral feeding and safe discharge are important goals of secondary treatment and defect closure was commonly applied to achieve these goals. Treatment strategies were determined by clinical findings at AL diagnosis. Although treatment goals were largely similar, preferred treatment principles and modalities varied widely.

The main strength of this study was the mixed-methods design, which is advocated to balance strengths and limitations of both methods.^33^ To our knowledge this is the first study to explore treatment of AL by combining quantitative and qualitative data. These did not only provide insight on the ‘how’, but also on the ‘why’: it has provided an elaborate overview of AL treatment and enabled further explanation and interpretation through the views of experts.

This study has some limitations. First, the results may not reflect every practice worldwide. Even though the survey was distributed globally, the majority of respondents were from Europe. In addition, the majority of experts were from Europe too, and no experts from Australia or Africa participated in the focus groups. Consequently, the practices of other continents are underrepresented in study outcomes. Second, the definition of defect closure may have been too broad as it included endoscopic techniques, surgical approaches and esophageal diversion. Although the physiological mechanism of these techniques is similar, i.e. to stop or prevent further leakage, different experts regarded esophageal diversion as a last resort, and thus may be recognized as a separate treatment principle. Third, the completion rate of the survey was 69% which is suboptimal and may reflect the length of the survey and difficulty of answering the case vignettes.^34^ Fourth, although the current study provides rationales for different treatment strategies, it did not address how long to persist with various treatment modalities or to assess when a certain approach is inappropriate and should be abandoned. In addition, the case vignettes did not include every clinical aspect that may determine the treatment strategy of AL. For example, airway fistula, defect size or configuration of fluid collections were not included. Still, these factors did not emerge as important determinants for overall treatment strategies during the focus groups and therefore, most important determinants were likely included. Finally, the current study specifically focused on surgeons. Although surgeons have a central role in management of patients with AL, future studies may include other physicians such as gastroenterologists and radiologists to investigate the multidisciplinary aspect of AL management.

The overall treatment strategy for AL was determined by the condition of the conduit and the presence of intrathoracic fluid collections. Consequently, three clinical subgroups may be distinguished, largely in line with previous literature^7^: patients with local symptoms (i.e. no intrathoracic collections), patients with intrathoracic collections and patients with overall conduit ischemia or necrosis. Firstly, patients with local symptoms may be treated successfully by supportive measures with antibiotics and/or feeding support with success.^35^ Nevertheless, many surgeons would still intervene by non-surgical measures to reduce the risk of sepsis. Secondly, in patients with intrathoracic collections, drainage may be routinely applied. In line with previous suggestions, pleural collections were seen as more severe than mediastinal collections especially in case of loculated pleural collections.^8^ Thirdly, patients with overall conduit ischemia or necrosis were thought to require resection of the affected tissue followed by a reconstruction of the anastomosis or diversion. Still, small proportions of ischemia/necrosis may be treated non-surgically, and successful stent treatment has been reported.^36^

Location of the anastomosis and presence of organ failure were found to influence the choice of treatment. Previous studies have separated treatment of cervical and intrathoracic leaks entirely.^7^^,^^37^^,^^38^ However, current findings indicate that the location of the anastomosis is one of multiple factors by which a strategy is determined and the treatment strategy of cervical and intrathoracic leaks has substantial overlap: there were no differences in goals of treatments and overall principles of treatment (e.g. drain fluid collections). Furthermore, strategies for treatment of cervical and intrathoracic leaks were comparable in case of intrathoracic fluid collections. Although some experts thought that treatment of cervical and intrathoracic leaks was different, others approached both leaks similarly. Even so, some differences were found: the threshold for drain placement/drainage may be lower in cervical leaks as this can be achieved more easily through cervical wound opening. In intrathoracic leaks, endoscopic or radiologic techniques may be required to achieve drainage, and thus the modalities used to achieve drainage differ between cervical and intrathoracic leaks. Moreover, defect closure by stent or endoVAC could be less feasible in cervical leaks, although successful cervical stent and endoVAC treatment have been reported.^39^^,^^40^ Regarding organ failure, patients with organ failure were treated surgically more often, as surgery was thought to provide swift control of sepsis. However, the role of surgical treatment may be ambiguous as surgery may also lead to a ‘second hit’ in critically ill patients.^41^

Treatment strategies of AL seem to be constantly evolving based on individual experiences and successes or failures of specific modalities. However, in absence of robust scientific evidence, these strategies do not seem to converge as the current study found a large variation in line with previous research.^7^ Rather than regional or intercontinental variation, treatment strategies seemed to vary from center to center within countries and continents. Although there is a need for guidelines, evidence for AL treatment is currently lacking and conducting a randomized clinical trial in patients with AL will be challenging.^1^^,^^35^ Still, our findings may aid development of consensus-based clinical guidelines: a first consensus statement may focus on general diagnostic and therapeutic principles that apply universally to management of AL.

Next to guideline development, future studies may aim to identify optimal treatment for the three identified clinical subgroups. In contrast with previous suggestions, the experts suggested that future studies could focus on treatment strategies rather than specific modalities.^42^ Firstly, in patients with local symptoms, non-interventional (i.e. supportive) treatment may be further investigated. Secondly, in patients with intrathoracic collections, the benefits of defect closure are yet to be properly evaluated. Although widely propagated in recent literature,^40^^,^^43^ the rationale of defect closure was much debated even amongst experts, and therefore the role of defect closure and especially stent or endoVAC remains unclear. Finally, in patients with conduit necrosis, future studies may explore outcomes of continuity-preserving versus direct-diversion strategies. Experts highlighted that even within these subgroups large variations in leak severity may exist. Future research may adjust for leak severity within subgroups by using the recently developed Severity of Esophageal Anastomotic Leak (SEAL) score, a tool to determine leak severity at diagnosis.^44^

In conclusion, this mixed-methods study showed that AL treatment strategies are determined by vitality of the gastric conduit and presence of intrathoracic fluid collections. Moreover, treatment modalities are chosen based on the location of the anastomosis (i.e. cervical or intrathoracic) and patient condition (e.g. organ failure). Still, there is a large variation in preferred principles and modalities. Future research should investigate the treatment strategy for different presentations of AL and may aim to reach consensus on optimal treatment for patients with AL after esophagectomy.

## Supplementary Material

Supplements_doac020
