# Supplementary material for: Treatment of anastomotic leak after esophagectomy: insights of an international case vignette survey and expert discussions
Source: Dis Esophagus. 2022 Apr 12;35(12):doac020. doi: 10.1093/dote/doac020 (PMC9753084; doi:10.1093/dote/doac020)
Supplement: Supplements_doac020 [file supplements_doac020.docx]

# Supplementary materials

[Supplementary materials 1](#_Toc94022842)

[eTable 1: Experts participating in the focus groups 2](#_Toc94022843)

[eTable 2: Quotes of participating experts during the focus groups 3](#_Toc94022844)

[eTable 3: Described treatment strategies of experts participating in the focus groups 5](#_Toc94022845)

[Supplement 1: Case Vignette Survey 7](#_Toc94022846)

[Supplement 2: Topic guide focus groups international experts 35](#_Toc94022847)

## eTable 1: Experts participating in the focus groups

| **Name** | **Center** | **Country** | **Continent** |
| --- | --- | --- | --- |
| **Elke van Daele** | Ghent University Hospital | Belgium | Europe |
| **Lorenzo Ferri** | McGill University Health Centre, Montreal General Hospital | Canada | North America |
| **Suzanne Gisbertz** | Amsterdam UMC, University of Amsterdam, Cancer Center Amsterdam | The Netherlands | Europe |
| **Ewen Griffiths** | University Hospitals Birmingham NHS Foundation Trust, Queen Elizabeth Hospital | United Kingdom | Europe |
| **Peter Grimminger** | University Medical Center Mainz | Germany | Europe |
| **George Hanna** | Imperial College, London | United Kingdom | Europe |
| **Michal Hubka** | Virginia Mason Medical Center, Seattle | United States of America | North America |
| **Simon Law** | Queen Mary Hospital, Hong Kong | China | Asia |
| **Donald Low** | Virginia Mason Medical Center, Seattle | United States of America | North America |
| **Misha Luyer** | Catharina Hospital, Eindhoven | The Netherlands | Europe |
| **Robert Merritt** | Ohio State University - Wexner Medical Center, Columbus | United States of America | North America |
| **Christopher Morse** | Massachusetts General Hospital, Boston | United States of America | North America |
| **Carmen Mueller** | McGill University Health Centre, Montreal General Hospital | Canada | North America |
| **Grard Nieuwenhuijzen** | Catharina Hospital, Eindhoven | The Netherlands | Europe |
| **Magnus Nilsson** | CLINTEC, Karolinska Institutet, Karolinska University Hospital | Sweden | Europe |
| **John Reynolds** | Trinity St. James's Cancer Institute | Ireland | Europe |
| **Ulysses Ribeiro** | University of Sao Paulo | Brazil | South America |
| **Riccardo Rosati** | San Raffaele Hospital IRCCS, Milan | Italy | Europe |
| **Yaxing Shen** | Zhongshan Hospital, Fudan University, Shanghai | China | Asia |
| **Bas Wijnhoven** | Erasmus University Medical Centre, Rotterdam | The Netherlands | Europe |

## eTable 2: Quotes of participating experts during the focus groups

| **Treatment strategies**  “We deal with people who are different, and we always treat patients individually. The treatment strategy comes first and then after strategy we apply a modality.” (expert, focus group 5)  “There is no general [treatment] strategy I think” (expert, focus group 4)  “It is always stunning for me to see how everyone approaches [anastomotic leak] differently, the wide variety of approaches.” (expert, focus group 3)  “Drainage is the most important, the dirty stuff needs to come out (…) If you need to put it in order, drainage always comes first. If you drain it adequately, you don’t really need to close the hole.” (expert, focus group 5)  “Generally, my approach has been conservative: giving tissue that will heal itself enough time to do that, support the patient and make sure that what can be drained is drained. I am not an advocate of stent placement and have not used endoVAC. (…) The goal is really to get the patient safely through it and I do not mind some extra period in hospital.” (expert, focus group 2)  “the reason for putting a drain in [during stent treatment] is the same reason we drain abscesses. We are taking an open infected space, and we are making it a closed infected space.” (expert, focus group 6)  “The problem with stent treatment is that you close off the cavity behind the leak, and thus you also need external drainage.” (expert, focus group 2)  “I think the principles [of stent and EsoSponge treatment] are very similar, regardless of whether it is a stent or a sponge. It is number one to control the leak, to drain the abscess and to prevent recurrence or re-accumulation, and to somehow feed the patient. To me it sounds like a very similar philosophy of care, just two different ways of going about it” (expert, focus group 6) |
| --- |
| **Fluid collections**  “Drainage is the key, and closure of the hole is secondary (…) because once I put in a stent, I have lost my access [for drainage] from the inside” (expert, focus group 5)  “If there is a collection, it needs to be drained” (expert, focus group 4)  “It needs to be drained to dryness” (expert, focus group 5) |
| **Cervical versus intrathoracic leaks**  “Even with the McKeown resection and cervical anastomosis, I find that the anastomosis ends up at the level of the thoracic inlet. And often, treatment of thoracic versus cervical anastomotic leaks is not that different because I worry about mediastinal contamination. In the vast majority [of patients], some sort of esophageal stent for 2 weeks is the most commonly used modality.” (expert, focus group 4)  “Most of our anastomoses are cervical, so [in case of a leak] we open the cervical wound for drainage. I agree with the previously mentioned principles [drainage], but because our anastomosis is made through the cervical incision, it is easier for us to perform drainage. We will consider stent placement, but do not perform stent placement often.” (expert, focus group 2)  “It is easy to open the cervical wound, so you would do that anyhow. If it is a contained leak in the neck or in the upper mediastinum, you may be able to open it via the neck wound. If it leaks down [intrathoracically] you would need a different approach for drainage, but conceptually it is still drainage. If we can drain intrathoracic leaks non-surgically, we do it endoscopically.” (expert, focus group 4) |
| **Conduit ischemia and necrosis**  “If you have massive necrosis of the gastric tube, you have to disconnect the anastomosis and remove the necrosis; (…) even if we have limited necrosis, we manage to [treat patients] with suction drainage.” (expert, focus group 2)  “If there is gastric conduit ischemia or necrosis, we may consider a revision of the anastomosis or even an esophageal diversion. (…) Necrosis is not salvageable, you definitely need to remove the stomach.” (expert, focus group 1)  “My experience is that small necrosis, you can stent or you can treat it using EsoSponge, we have been doing that. If it is large necrosis it is a totally different issue and septic patients with more than 5cm necrosis we would probably deviate. But in patients with small necrosis, we treat them the same way we treat other patients and usually it works.” (expert, focus group 1) |
| **Multiorgan failure**  “Patients with sepsis and without, those are two clinically different scenarios that require different treatment.” (expert, focus group 1)  “If you do a thoracotomy, you probably deal with a problem at an earlier stage and the patient is in recovery quicker. If you drain it really well, do a thoracotomy it is probably better. The patient recovers quicker and you do not need many interventions. Percutaneous drainage is not always ideal.” (expert, focus group 4) |
| **Other**  “Diagnosis of anastomotic leakage has become more aggressive, while treatment of anastomotic leakage has become more conservative” (expert, focus group 1)  “Our modality of diagnosis usually is esophagram, followed secondarily by a CT-scan of the chest with water-soluble oral contrast and subsequent endoscopy in every case. We have detected small leaks on endoscopy that we were unable to detect using [esophagram or CT-scan] on a number of occasions.” (expert, focus group 4)  “I cannot tell you what I usually do. I can tell you that as a starting point, we always start with CT and endoscopy, because both are important with respect to making decisions.” (expert, focus group 6)  “I really am intrigued by the idea of covering versus not covering the defect. I don’t think drainage is a controversial issue (…) but I think that the coverage is a major topic.” (expert, focus group 4)  “In anastomotic leak it is not an issue of where we do the anastomosis. If we do a study on anastomotic leak, we can do subgroup analysis on the cervical and the intrathoracic, but the main topic should be focused on anastomotic leak, not divided in two parts.” (expert, focus group 2)  “The big thing is the overtreatment of minor leaks and the under- or inappropriate treatment of the major leaks. Those are the areas that need standardization.” (expert, focus group 2) |

Abbreviations: CT, Computed Tomography; endoVAC, endoscopic vacuum-assisted closure

## eTable 3: Described treatment strategies of experts participating in the focus groups

| **Described general treatment strategy ^a^** |
| --- |
| Routine placement of endoVAC, mostly placed intraluminally. Endoscopic drainage is used for small leaks. Conservative treatment for asymptomatic patients. |
| Drainage and stent placement for patients with a viable conduit. Diversion in case of overall ischemia or necrosis. |
| Conservative treatment for patients with contained leaks and without severe illness. Radiological drainage for undrained collections. Endoscopic stent placement after endoscopic wash-out for patients with systemic illness. Reoperation with drainage and revision of the anastomosis for patients with loculated pleural fluid collections. |
| Nasogastric suction tube (sometimes placed through the defect). Surgical disconnection for patients with organ failure or necrosis. Stent after successful drainage in some patients. |
| Routine placement of two drains, cervical drain and chest drain; Simple drainage in patients with good lung expansion. |
| Drain what can be drained, support patient to allow time to heal the leak. Small leaks treated conservatively. |
| In absence of ischemia or necrosis, endoVAC was routinely used, intraluminal for small leaks, extraluminal for large leaks. Endoscopic suction drain placement through the defect would be used for moderate sized leaks. Reoperation with reconstruction or diversion for patients with sepsis or conduit necrosis. |
| Stent placement and radiological drainage for patients without organ failure. Surgical wash-out of chest and revision/repair of the anastomosis for patients with organ failure. Esophageal diversion for most critical patients. |
| Most patients are treated conservatively (i.e. antibiotics and non-surgical drainage). Surgery to achieve complete drainage if non-surgical drainage was not feasible, no routine defect closure (revision/repair of anastomosis) during reoperation. |
| Algorithmic treatment strategy: esophageal diversion for patients with necrosis; stent placement and percutaneous/endoscopic drainage for patients with extensive contamination but viable conduit. |
| Drainage through opening of cervical wound in the majority of patients. Surgical treatment for patients with intrathoracic (i.e. mediastinal or pleural) manifestations to achieve optimal drainage. |
| Strategy based on severity of sepsis. Drainage to control sepsis and defect closure if possible; Wound opening for cervical leaks. Radiological/surgical drainage combined with stent placement for patients with intrathoracic leak and sepsis. Revision of conduit for hemodynamically stable patients with limited ischemia/necrosis, esophageal diversion in case of overall ischemia/necrosis. |
| Control sepsis through complete drainage. Conservative treatment for patients with small leak without contaminated cavities. EndoVAC for patients with mediastinal collections. Multiple percutaneous drains for pleural collections. Defect closure using stent placement for some patients, especially in a larger defect. Esophageal diversion for complete necrosis, stent placement for limited necrosis in case of systemic illness. Observation in case of limited necrosis in clinically-well patients. |
| Routine stent placement, even for patients with limited necrosis. Radiological drainage or endoscopic wash-out before stent placement in patients with mediastinal collections. Surgical drainage in case of sepsis. Revision of conduit and anastomosis in case of extensive necrosis, esophageal diversion for total necrosis. |
| EndoVAC for patients without ischemia or necrosis. Additional radiological drainage in case of pleural or large mediastinal collection. Surgical repair of anastomosis using muscle flap in case of salvageable necrosis, esophageal diversion for extensive necrosis. |
| Conservative treatment for patients without systemic signs of illness. Stent and endoVAC treatment in patients without hemodynamic instability: endoVAC in case of a dependent cavity (i.e. a cavity where fluid will flow in but not flow out with the patient upright). Non-interventional management for non-dependent cavities (i.e. a cavity where swallowed material will flow in and out with gravity). Surgical drainage in patients with organ failure. Esophageal diversion as a last resort for patients with extensive transmural necrosis of the conduit. |

^a^ The listed strategies are those that were described in detail during the focus groups. The strategy of some experts may not be included if not described in detail during the focus groups. Abbreviations: endoVAC; endoscopic vacuum-assisted closure device;

## Supplement 1: Case Vignette Survey

Survey Introduction

Treatment of Anastomotic Leakage Survey

This survey is aimed to gain insight in the treatment of patients with anastomotic leakage after esophagectomy. The survey consists of 2 parts: a short questionnaire and 10 clinical cases. It will take approximately 15 minutes to complete the whole survey.

All responses will be analyzed and reported anonymously. The results of the survey will be prepared for publication with the aim to aid clinical decision making and to guide development and analysis of future studies focused on treatment of anastomotic leakage.

Note: this survey is not focused at changes or developments during COVID-19. Please answer the questions as before/regardless of the pandemic.

* 1. Are you a surgeon performing esophagectomy for patients with esophageal cancer?


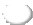

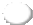
 Yes No

Part 1 - Questionnaire

Treatment of Anastomotic Leakage Survey

- 2. In which country do you work?
  - 3. Which of the following hospital types best describes the hospital your work in?


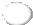
 General hospital, non-teaching
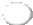
 General hospital, teaching


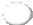
 University hospital
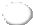
 Other (please specify)

- 4. How many years of experience do you have with esophageal resections for cancer?


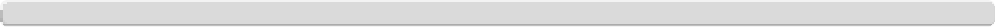

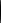

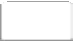

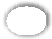

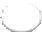


0

25

50

- 5. Approximately how many esophageal resections have you performed as a surgeon?


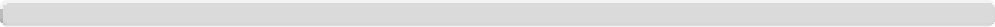

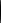

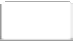

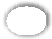

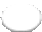


1

100

200+

- 6. How many esophagectomies are performed annually in your hospital?


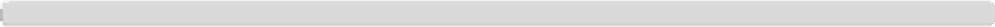

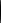

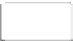

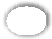

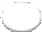


1

100

200+

- - 7. What is your most commonly applied location for the anastomosis (in case of a distal esophageal tumor)?


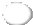
 Intrathoracic anastomosis
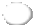
 Cervical anastomosis

- - 8. What is your most commonly applied surgical approach for esophagectomy?


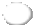

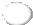
 Transhiatal
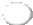
 Transthoracic

Other (please specify)

- - 9. What is your most commonly applied surgical technique?


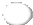
 Open esophagectomy


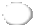
 Hybrid esophagectomy, laparoscopic
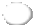
 Hybrid esophagectomy, thoracoscopic


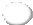
 Totally minimally invasive esophagectomy (TMIE)


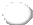
 Robotic-assisted minimally invasive esophagectomy (RAMIE)

- - 10. Do you routinely perform drain placement during esophageal resection? (if yes, please describe drain placement locations)


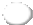
 Yes
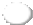
 No

If yes, please specify the location of the routinely placed drains during esophagectomy;

- - 11. What is your most commonly performed feeding access during/after esophagectomy?


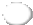
 Feeding jejunostomy


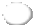
 Nasojejunal/Nasoduodenal feeding tube
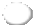
 Total Parenteral Nutrition


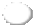
 IV fluids only


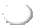

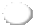
 Direct oral feeding
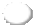
 None

Other (please specify)

Part 1 - Questionnaire

Treatment of Anastomotic Leakage Survey

- - 12. Do you have a local treatment protocol for anastomotic leakage after esophagectomy at your hospital?


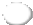
 Yes
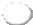
 No

- 13. Which treatment goals are you aiming to achieve when treating a patient with anastomotic leakage? Please rank the treatment goals from most (1) to least important (5);


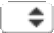
´

Reducing hospital length of stay


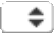
´

Prevention or reduction of morbidity


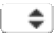
´

Reduction of costs


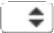
´

Prevention of mortality


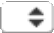
´

Maintaining Quality of Life

- - 14. Please check **all** therapeutic modalities **available** in your hospital:

Ultrasound guided drainage CT-scan guided drainage Endoscopic drainage Endoscopic stent placement

Endoscopic vacuum assisted closure device (e.g. endoVAC/esosponge) Endoscopic Clipping

Scopic surgical treatment (laparoscopy, thoracoscopy, video-assisted thoracoscopy) Open surgical treatment (cervicotomy, laparotomy, thoracotomy)

Other (please specify)

- - 15. What would best describe your routine treatment strategy in patients with anastomotic leakage?


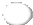
 Direct surgical intervention


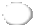
 Conservative or minimally invasive step-up
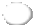
 Other

Please describe your routine treatment strategy in a few sentences

- - 16. Is **antibiotic** therapy indicated routinely in patients with anastomotic leakage?


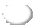
 Yes
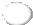
 No

- - 17. Is **antifungal** therapy indicated routinely in patients with anastomotic leakage?


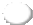
 Yes
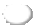
 No

- - 18. Do you think there is a fundamental difference in the treatment of patients with cervical anastomotic leakage after transthoracic (McKeown) vs. transhiatal (Orringer) esophagectomy?


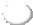
 Yes
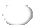
 No

If you answered "yes", please explain the difference

- - 19. Do you think there is a fundamental difference in the treatment of patients with cervical anastomotic leakage vs. intrathoracic leakage after transthoracic (McKeown or Ivor Lewis) esophagectomy?


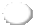
 Yes
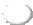
 No

If you answered "yes", please explain the difference

- - 20. Patients with anastomotic leakage and transmural necrosis of the gastric conduit are often regarded as a different clinical group as opposed to patients with anastomotic leakage with an overall well-perfused gastric conduit. How do you treat patients with anastomotic leakage and an ischemic gastric conduit?


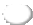
 Similar to patients with anastomotic leakage with a well-perfused conduit
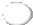
 Similar to patients with gastric conduit necrosis


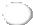
 Separate clinical group

Please give a comment on your choice

- - 21. What is your dietary prescription for patients with anastomotic leakage?


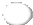

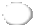

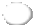

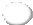

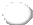

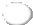

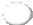

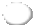

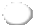


No restrictions (full diet) Liquids

Only water Nil per mouth

Dependent on the leakage characteristics

If dependent on the leakage characteristics, please summarize what characteristics indicate any dietary prescription

- - 22. If indicated, what is your preferred route for nutritional support for patients with anastomotic leakage?

Enteral, nasojejunal feeding tube Enteral, surgical jejunostomy Parenteral (TPN)

Other

Part 2: Clinical Cases

Treatment of Anastomotic Leakage Survey

In this second part of the questionnaire 10 short clinical cases will be presented. The cases include a short clinical description and a CT or endoscopy image to illustrate the clinical presentation. All patients are 60 years old without significant comorbidities, with anastomotic leakage confirmed by CT-scan and endoscopy after esophagectomy with gastric tube reconstruction for esophageal cancer. Other complications or diseases (e.g. pneumonia, urinary tract infection) as a cause of the symptoms have been ruled out.

Three distinct mechanisms in treatment of anastomotic leakage can be distinguished:

*Drainage*: interventions aimed to drain extraluminal leakage fluids, fluid collections and/or contaminated cavity’s, e.g. endoscopic drainage, opening of a wound, chest tubes placement, radiological drainage and/or surgical drainage;

*Defect closure:* interventions aimed to close the defect and prevent further leakage of fluids,

e.g. stent placement, endoVAC (also drainage), surgical stitching, re-anastomosis, muscle flap repair or esophageal diversion.

*Supportive interventions*: aimed to support the patient during anastomotic leakage, e.g. dietary restrictions, antibiotics and/or feeding support;

In all 10 cases you will be asked to answer the same questions:

1. What treatment mechanism(s) would you choose as *initial* treatment for this patient? (multiple options possible)
2. Which modality would you choose to effectuate *chosen mechanism(s)* in this patient, with the modalities available at your center?

**Please read the cases carefully before answering the questions.**

Case A

Treatment of Anastomotic Leakage Survey

Day 5 after esophagectomy with cervical anastomosis, admitted to the surgical ward

Patient with fever and discomfort, CRP of 220mg/L and leukocytosis, no signs of organ failure

CT shows contrast leakage without cervical fluid collections, there are no pleural or mediastinal fluid collections

Endoscopy shows no signs of gastric conduit necrosis There are no post-operative drains in situ


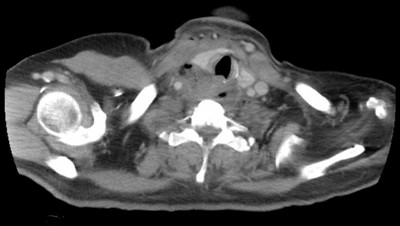


- - 23. What treatment mechanism(s) would you choose as initial treatment for this patient? (multiple options possible)

Drainage Defect closure

Supportive interventions

1. If you chose "Drainage" in question 23:

Which modality would you choose to effectuate drainage in this patient, with the modalities available at your center?


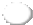
 Bedside opening of the wound


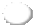
 Radiological drainage (CT- or ultrasound guided)
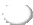
 Chest tube drainage


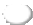
 Endoscopic drainage (NG tube near defect or endoscopic drain through defect)
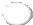
 Surgical drainage (cervicotomy, thoracotomy and/or thoracoscopy)


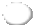

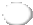
 Other (please specify)

Not applicable

1. If you chose "Defect closure" in question 23:

Which modality would you choose to effectuate defect closure in this patient, with the modalities available at your center?


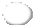
 Endoscopic stent placement
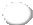
 EndoVAC


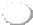
 Surgical closure (suturing, re-anastomosis, flap repair)
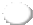
 Disconnection and esophageal diversion


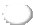
 Other (please specify)


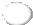
 Not applicable

1. If you chose "Supportive interventions" in question 23:

Which modalities would you choose to effectuate supportive care in this patient, with the modalities available at your center? (multiple options possible)

Dietary restrictions Antibiotic treatment

Feeding support (either enteral or parenteral) Not applicable

Case B

Treatment of Anastomotic Leakage Survey

Day 5 after esophagectomy with cervical anastomosis, admitted to the surgical ward

Patient suffers from fever and discomfort, CRP of 220mg/L and leukocytosis, no signs of organ failure

CT shows contrast leakage **with** a cervical fluid collection, there are no pleural or mediastinal fluid collections

Endoscopy shows no signs of gastric conduit necrosis There are no post-operative drains in situ


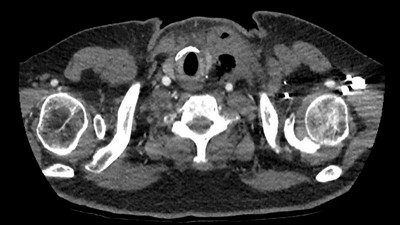


- - 27. What treatment mechanism(s) would you choose as initial treatment for this patient? (multiple options possible)

Drainage Defect closure

Supportive interventions

1. If you chose "Drainage" in question 27:

Which modality would you choose to effectuate drainage in this patient, with the modalities available at your center?


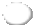
 Bedside opening of the wound


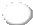
 Radiological drainage (CT- or ultrasound guided)
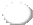
 Chest tube drainage


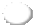
 Endoscopic drainage (NG tube near defect or endoscopic drain through defect)
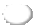
 Surgical drainage (cervicotomy, thoracotomy and/or thoracoscopy)


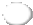

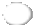
 Other (please specify)

Not applicable

1. If you chose "Defect closure" in question 27:

Which modality would you choose to effectuate defect closure in this patient, with the modalities available at your center?


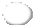
 Endoscopic stent placement
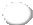
 EndoVAC


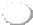
 Surgical closure (suturing, re-anastomosis, flap repair)
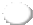
 Disconnection and esophageal diversion


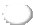
 Other (please specify)


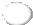
 Not applicable

1. If you chose "Supportive interventions" in question 27:

Which modalities would you choose to effectuate supportive care in this patient, with the modalities available at your center? (multiple options possible)

Dietary restriction Antibiotic treatment

Feeding support (either enteral or parenteral) Not applicable

Case C

Treatment of Anastomotic Leakage Survey

Day 5 after esophagectomy with **intrathoracic** anastomosis, admitted to the surgical ward Patient with fever and discomfort, CRP of 220mg/L and leukocytosis, no signs of organ failure CT shows contrast leakage **without** pleural or mediastinal fluid collections

Endoscopy shows no signs of gastric conduit necrosis There are no post-operative drains in situ


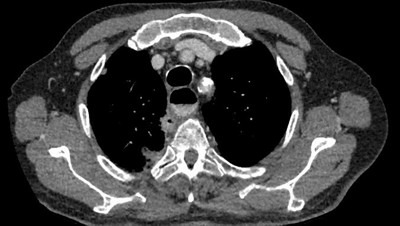


- - 31. What treatment mechanism(s) would you choose as initial treatment for this patient? (multiple options possible)

Drainage Defect closure

Supportive interventions

1. If you chose "Drainage" in question 31:

Which modality would you choose to effectuate drainage in this patient, with the modalities available at your center?


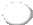
 Bedside opening of the wound

Radiological drainage (CT- or ultrasound guided) Chest tube drainage

Endoscopic drainage (NG tube near defect or endoscopic drain through defect) Surgical drainage (cervicotomy, thoracotomy and/or thoracoscopy)

Other (please specify)

Not applicable

1. If you chose "Defect closure" in question 31:

Which modality would you choose to effectuate defect closure in this patient, with the modalities available at your center?

Endoscopic stent placement EndoVAC

Surgical closure (suturing, re-anastomosis, flap repair) Disconnection and esophageal diversion

Other (please specify)

Not applicable

1. If you chose "Supportive interventions" in question 31:

Which modalities would you choose to effectuate supportive care in this patient, with the modalities available at your center? (multiple options possible)

Dietary restriction Antibiotic treatment

Feeding support (either enteral or parenteral) Not applicable

Case D

Treatment of Anastomotic Leakage Survey

Day 5 after esophagectomy with intrathoracic anastomosis, admitted to the surgical ward Patient with fever and discomfort, CRP of 220mg/L and leukocytosis, no signs of organ failure CT shows contrast leakage **with a mediastinal fluid collection**, but no pleural fluid collections Endoscopy shows no signs of gastric conduit necrosis

There are no post-operative drains in situ

- - 35. What treatment mechanism(s) would you choose as initial treatment for this patient? (multiple options possible)

Drainage Defect closure

Supportive interventions

1. If you chose "Drainage" in question 35:

Which modality would you choose to effectuate drainage in this patient, with the modalities available at your center?

Bedside opening of the wound

Radiological drainage (CT- or ultrasound guided) Chest tube drainage

Endoscopic drainage (NG tube near defect or endoscopic drain through defect) Surgical drainage (cervicotomy, thoracotomy and/or thoracoscopy)

Other (please specify)

Not applicable

1. If you chose "Defect closure" in question 35:

Which modality would you choose to effectuate defect closure in this patient, with the modalities available at your center?

Endoscopic stent placement EndoVAC

Surgical closure (suturing, re-anastomosis, flap repair) Disconnection and esophageal diversion

Other (please specify)

Not applicable

1. If you chose "Supportive interventions" in question 35:

Which modalities would you choose to effectuate supportive care in this patient, with the modalities available at your center? (multiple options possible)

Dietary restriction Antibiotic treatment

Feeding support (either enteral or parenteral) Not applicable

Case E

Treatment of Anastomotic Leakage Survey

Day 5 after esophagectomy with intrathoracic anastomosis, admitted to the surgical ward Patient with fever and discomfort, CRP of 220mg/L and leukocytosis, no signs of organ failure

CT shows contrast leakage **with a contaminated pleural fluid collection, but no mediastinal fluid collections**

Endoscopy shows no signs of gastric conduit necrosis There are no post-operative drains in situ

- - 39. What treatment mechanism(s) would you choose as initial treatment for this patient? (multiple options possible)

Drainage Defect closure

Supportive interventions

1. If you chose "Drainage" in question 39:

Which modality would you choose to effectuate drainage in this patient, with the modalities available at your center?

Bedside opening of the wound

Radiological drainage (CT- or ultrasound guided) Chest tube drainage

Endoscopic drainage (NG tube near defect or endoscopic drain through defect) Surgical drainage (cervicotomy, thoracotomy and/or thoracoscopy)

Other (please specify)

Not applicable

1. If you chose "Defect closure" in question 39:

Which modality would you choose to effectuate defect closure in this patient, with the modalities available at your center?

Endoscopic stent placement EndoVAC

Surgical closure (suturing, re-anastomosis, flap repair) Disconnection and esophageal diversion

Other (please specify)

Not applicable

1. If you chose "Supportive interventions" in question 39:

Which modalities would you choose to effectuate supportive care in this patient, with the modalities available at your center? (multiple options possible)

Dietary restriction Antibiotic treatment

Feeding support (either enteral or parenteral) Not applicable

Case F

Treatment of Anastomotic Leakage Survey

Day 5 after esophagectomy with intrathoracic anastomosis, admitted to the surgical ward Patient with fever and discomfort, CRP of 220mg/L and leukocytosis, no signs of organ failure

CT shows contrast leakage **with a mediastinal fluid collection, and with a contaminated pleural fluid collection**

Endoscopy shows no signs of gastric conduit necrosis There are no post-operative drains in situ

- - 43. What treatment mechanism(s) would you choose as initial treatment for this patient? (multiple options possible)

Drainage Defect closure

Supportive interventions

1. If you chose "Drainage" in question 43:

Which modalities would you choose to effectuate drainage in this patient, with the modalities available at your center?

Bedside opening of the wound

Radiological drainage (CT- or ultrasound guided)

Chest tube drainage

Endoscopic drainage (NG tube near defect or endoscopic drain through defect)

Surgical drainage (cervicotomy, thoracotomy and/or thoracoscopy)

Drainage not applicable

Mediastinal collection

Pleural collection Other (please specify)

1. If you chose "Defect closure" in question 43:

Which modality would you choose to effectuate defect closure in this patient, with the modalities available at your center?

Endoscopic stent placement EndoVAC

Surgical closure (suturing, re-anastomosis, flap repair) Disconnection and esophageal diversion

Other (please specify)

Not applicable

1. If you chose "Supportive interventions" in question 43:

Which modalities would you choose to effectuate supportive care in this patient, with the modalities available at your center? (multiple options possible)

Dietary restriction Antibiotic treatment

Feeding support (either enteral or parenteral) Not applicable

Case G

Treatment of Anastomotic Leakage Survey

Day 5 after esophagectomy with intrathoracic anastomosis, admitted to the surgical ward Patient with fever and discomfort, CRP of 220mg/L and leukocytosis, no signs of organ failure

CT shows contrast leakage with a mediastinal fluid collection, and with a contaminated pleural fluid collection

There are mediastinal and pleural post-operative drains in situ which drain the collections inadequately

Endoscopy shows no signs of gastric conduit necrosis

* 47. What treatment mechanism(s) would you choose as initial treatment for this patient? (multiple options possible)

Drainage Defect closure

Supportive interventions

1. If you chose "Drainage" in question 47:

Which modalities would you choose to effectuate drainage in this patient, with the modalities available at your center?

Bedside opening of the wound

Radiological drainage (CT- or ultrasound guided)

Chest tube drainage

Endoscopic drainage (NG tube near defect or endoscopic drain through defect)

Surgical drainage (cervicotomy, thoracotomy and/or thoracoscopy)

Drainage not applicable

Mediastinal collection

Pleural collection Other (please specify)

1. If you chose "Defect closure" in question 47:

Which modality would you choose to effectuate defect closure in this patient, with the modalities available at your center?

Endoscopic stent placement EndoVAC

Surgical closure (suturing, re-anastomosis, flap repair) Disconnection and esophageal diversion

Other (please specify)

Not applicable

1. If you chose "Supportive interventions" in question 47:

Which modalities would you choose to effectuate supportive care in this patient, with the modalities available at your center? (multiple options possible)

Dietary restriction Antibiotic treatment

Feeding support (either enteral or parenteral) Not applicable

Case H

Treatment of Anastomotic Leakage Survey

Day 5 after esophagectomy with intrathoracic anastomosis, admitted to the **intensive care unit** Patient with fever and discomfort, CRP of 220mg/L and leukocytosis, patient **does require mechanic ventilation and inotropic support**

CT shows contrast leakage with a mediastinal fluid collection, and with a contaminated pleural fluid collection

Endoscopy shows no signs of gastric conduit necrosis There are **no post-operative drains in situ**

* 51. What treatment mechanism(s) would you choose as initial treatment for this patient? (multiple options possible)

Drainage Defect closure

Supportive interventions

1. If you chose "Drainage" in question 51:

Which modalities would you choose to effectuate drainage in this patient, with the modalities available at your center?

Bedside opening of the wound

Radiological drainage (CT- or ultrasound guided)

Chest tube drainage

Endoscopic drainage (NG tube near defect or endoscopic drain through defect)

Surgical drainage (cervicotomy, thoracotomy and/or thoracoscopy)

Drainage not applicable

Mediastinal collection

Pleural collection Other (please specify)

1. If you chose "Defect closure" in question 51:

Which modality would you choose to effectuate defect closure in this patient, with the modalities available at your center?

Endoscopic stent placement EndoVAC

Surgical closure (suturing, re-anastomosis, flap repair) Disconnection and esophageal diversion

Other (please specify)

Not applicable

1. If you chose "Supportive interventions" in question 51:

Which modalities would you choose to effectuate supportive care in this patient, with the modalities available at your center? (multiple options possible)

Dietary restriction Antibiotic treatment

Feeding support (either enteral or parenteral) Not applicable

Case I

Treatment of Anastomotic Leakage Survey

Day 5 after esophagectomy with **cervical** anastomosis, admitted to the **surgical ward**

Patient with fever and discomfort, CRP of 220mg/L and leukocytosis, **no signs of organ failure** CT shows contrast leakage with a mediastinal fluid collection, and with a contaminated pleural fluid collection, there are no cervical fluid collections

Endoscopy shows no signs of gastric conduit necrosis There are no post-operative drains in situ

* 55. What treatment mechanism(s) would you choose as initial treatment for this patient? (multiple options possible)

Drainage Defect closure

Supportive interventions

1. If you chose "Drainage" in question 51:

Which modalities would you choose to effectuate drainage in this patient, with the modalities available at your center?

Bedside opening of the wound

Radiological drainage (CT- or ultrasound guided)

Chest tube drainage

Endoscopic drainage (NG tube near defect or endoscopic drain through defect)

Surgical drainage (cervicotomy, thoracotomy and/or thoracoscopy)

Drainage not applicable

Mediastinal collection

Pleural collection Other (please specify)

1. If you chose "Defect closure" in question 55:

Which modality would you choose to effectuate defect closure in this patient, with the modalities available at your center?

Endoscopic stent placement EndoVAC

Surgical closure (suturing, re-anastomosis, flap repair) Disconnection and esophageal diversion

Other (please specify)

Not applicable

1. If you chose "Supportive interventions" in question 55:

Which modalities would you choose to effectuate supportive care in this patient, with the modalities available at your center? (multiple options possible)

Dietary restriction Antibiotic treatment

Feeding support (either enteral or parenteral) Not applicable

Case J

Treatment of Anastomotic Leakage Survey

Day 5 after esophagectomy with cervical anastomosis, admitted to the **intensive care unit** Patient with fever and discomfort, CRP of 220mg/L and leukocytosis, patient **does require mechanic ventilation and inotropic support**

CT shows contrast leakage **with a mediastinal fluid collection**, but without pleural fluid collections

Endoscopy reveals transmural necrosis of the gastric conduit

There are no post-operative drains in situ

* 59. What treatment mechanism(s) would you choose as initial treatment for this patient? (multiple options possible)

Drainage

Defect closure (including esophageal diversion) Supportive interventions

1. If you chose "Drainage" in question 59:

Which modality would you choose to effectuate drainage in this patient, with the modalities available at your center?

Bedside opening of the wound

Radiological drainage (CT- or ultrasound guided) Chest tube drainage

Endoscopic drainage (NG tube near defect or endoscopic drain through defect) Surgical drainage (cervicotomy, thoracotomy and/or thoracoscopy)

Not applicable

Other (please specify)

1. If you chose "Defect closure" in question 59:

Which modality would you choose to effectuate defect closure in this patient, with the modalities available at your center?

Endoscopic stent placement EndoVAC

Surgical closure (suturing, re-anastomosis, flap repair) Disconnection and esophageal diversion

Not applicable

Other (please specify)

1. If you chose "Supportive interventions" in question 59:

Which modalities would you choose to effectuate supportive care in this patient, with the modalities available at your center? (multiple options possible)

Dietary restriction Antibiotic treatment

Feeding support (either enteral or parenteral) Not applicable

Survey complete

Treatment of Anastomotic Leakage Survey

**Thank you for your participation in our survey on treatment of anastomotic leakage after esophagectomy!**

**The results of the survey will be analyzed and published shortly.**

**If you have any remarks, please feel free to contact us through email by emailing Sander Ubels, coordinating investigator of the TENTACLE - Esophagus study (**[**sander.ubels@radboudumc.n**](mailto:%20sander.ubels@radboudumc.nl)**l) or visit our website** [**https://www.tentaclestudy.com**](https://www.tentaclestudy.com/)**/.**

## Supplement 2: Topic guide focus groups international experts

**General aim:** To gain insight in the choices that experts make when treating patients with anastomotic leakage after esophagectomy with gastric tube reconstruction.

**1. General introduction**

- Welcome the experts

- Introduction round

**2. Discussion**

**Part 1: clinical treatment strategy**

- General strategy
  What is your general strategy for treatment of patients with anastomotic leakage?

*Ask the others
Zoom in on reason for differences!*

*Ask why! Promote discussion!*

- Drainage

When do you choose to perform drainage?

*Why?*

*What are your (physiological) goals?*

*Which modality? When surgical?*

- Defect closure

What indicates defect closure?

*Why?*

*What are your physiological goals?*

*When do you choose which modality?*

*When do you choose disconnection?*

- Cervical vs. Intrathoracic anastomotic leaks

Are there differences? Could you describe the differences?

*Different physiological goals?*

*Different treatment goals?*

- Patient condition: organ failure

Is the approach to patients with organ failure different?

*Why is there a difference, is there a different treatment goal?*

*How do you interpret these findings?*

- Supportive interventions

When do you prescribe feeding support?

*Which method?*

What dietary prescription do you choose?

*And why?*

- Gastric conduit ischemia/necrosis

Is the approach to patients with gastric tube ischemia/necrosis different?

*Why?*

*Different physiological goals?*

*Ischemic: same as well-perfused or necrotic?*

**Part 2: anastomotic leakage research**

- Clinically relevant patient subgroups

Is there a need to define clinically relevant subgroups?

*How should these be created?*

*Would you analyze patients with cervical/intrathoracic anastomosis together or separately?*

*Would you analyze patients with undrained intrathoracic fluid collections separately?*

*Would you analyze patients with conduit necrosis separately? And patients with ischemia?*

- Analysis of treatments

How should we categorize the different leak treatments?

*In research would you focus on principle (i.e. closure/drainage) or on modality (i.e. stent/esosponge)?*

*What specific comparisons would you like to read about?*

**3. Wrap-up**

- Introduce and explain member check procedure

- Thank for participation!
